# Poly[bis­(1*H*-imidazole)bis­(μ_2_-1*H*-imidazolido)bis­(μ_2_-7-oxabicyclo­[2.2.1]heptane-2,3-dicarboxyl­ato)trizinc(II)]

**DOI:** 10.1107/S1600536810017915

**Published:** 2010-05-22

**Authors:** Qiu-Yue Lin, Na Wang, Yi-Zhou Wu

**Affiliations:** a Zhejiang Key Laboratory for Reactive Chemistry on Solid Surfaces, Institute of Physical Chemistry, Zhejiang Normal University, Jinhua, Zhejiang 321004, People’s Republic of China; b College of Chemistry and Life Science, Zhejiang Normal University, Jinhua 321004, Zhejiang, People’s Republic of China; c College of Public Administration, Zhejiang University, Hangzhou, Zhejiang 310027, People’s Republic of China

## Abstract

The title polymer, [Zn_3_(C_8_H_8_O_5_)_2_(C_3_H_3_N_2_)_2_(C_3_H_4_N_2_)_2_]_*n*_, was formed by the reaction of zinc acetate with imidazole and 7-oxabicyclo­[2.2.1]heptane-2,3-dicarboxylic anhydride (norcan­tharidine). One of the two crystallographically unique Zn^II^ atoms is four-coordinated by three N atoms of three imidazole ligands, two of which are deprotonated, and by one carboxyl­ate O atom of the demethyl­cantharate anion. The second Zn^II^ atom is situated on an inversion centre and is six-coordinated by the bridging O atoms of two symmetry-related demethyl­cantharate anions and by four carboxyl­ate O atoms of the corresponding carboxyl­ate groups. The polymeric crystal structure is additionally stabilized by N—H⋯O hydrogen bonding between the imidazole ligands and carboxyl­ate O atoms.

## Related literature

7-Oxabicyclo­[2.2.1]heptane-2,3-dicarboxylic anhydride (nor­can­tharidin) is a lower toxicity anti­cancer drug, see: Shimi *et al.* (1982 ▶). For cobalt complexes of norcantharidin, see: Wang *et al.* (1988 ▶) and for those including imidazole ligands, see: Furenlid *et al.* (1986 ▶); Zhu *et al.* (2003 ▶).
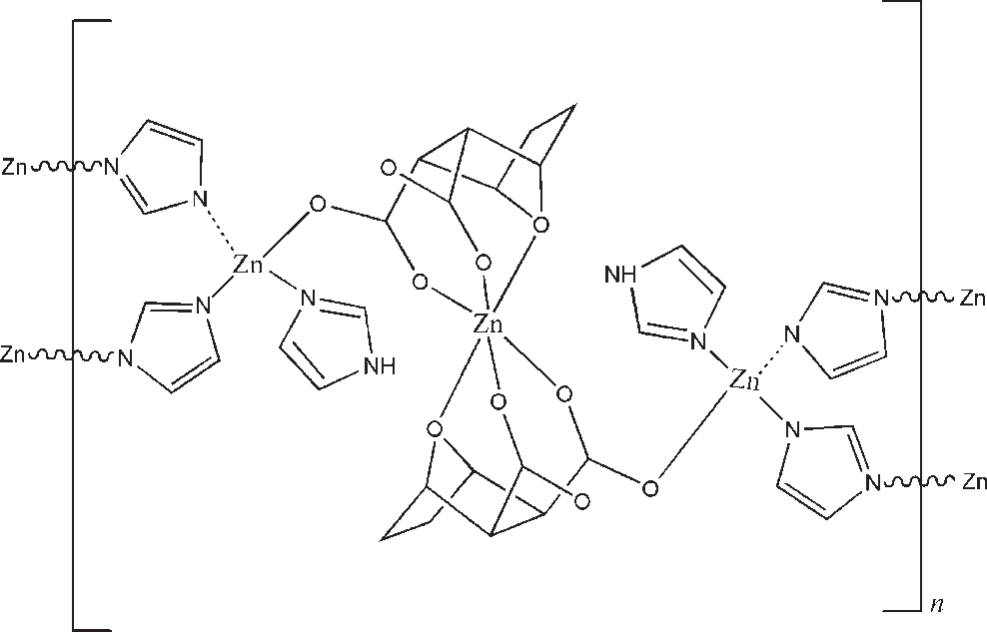


## Experimental

### 

#### Crystal data

[Zn_3_(C_8_H_8_O_5_)_2_(C_3_H_3_N_2_)_2_(C_3_H_4_N_2_)_2_]
*M* *_r_* = 834.71
Monoclinic, 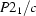

*a* = 7.9993 (1) Å
*b* = 22.3923 (2) Å
*c* = 9.7586 (1) Å
β = 112.633 (1)°
*V* = 1613.37 (3) Å^3^
*Z* = 2
Mo *K*α radiation
μ = 2.28 mm^−1^
*T* = 296 K
0.28 × 0.17 × 0.09 mm

#### Data collection

Bruker APEXII CCD diffractometer
Absorption correction: multi-scan (*SADABS*; Sheldrick, 1996 ▶) *T*_min_ = 0.629, *T*_max_ = 0.822
13439 measured reflections
3714 independent reflections
3197 reflections with *I* > 2σ(*I*)
*R*_int_ = 0.023

#### Refinement

*R*[*F* ^2^ > 2σ(*F* ^2^)] = 0.024
*wR*(*F* ^2^) = 0.060
*S* = 1.03
3714 reflections
223 parameters
H-atom parameters constrained
Δρ_max_ = 0.36 e Å^−3^
Δρ_min_ = −0.28 e Å^−3^

### 

Data collection: *APEX2* (Bruker, 2006 ▶); cell refinement: *SAINT* (Bruker, 2006 ▶); data reduction: *SAINT*; program(s) used to solve structure: *SHELXS97* (Sheldrick, 2008 ▶); program(s) used to refine structure: *SHELXL97* (Sheldrick, 2008 ▶); molecular graphics: *SHELXTL* (Sheldrick, 2008 ▶); software used to prepare material for publication: *SHELXL97*.

## Supplementary Material

Crystal structure: contains datablocks I, global. DOI: 10.1107/S1600536810017915/wm2332sup1.cif

Structure factors: contains datablocks I. DOI: 10.1107/S1600536810017915/wm2332Isup2.hkl

Additional supplementary materials:  crystallographic information; 3D view; checkCIF report

## Figures and Tables

**Table 1:** Selected bond lengths (Å)

Zn1—O2	1.9570 (12)
Zn1—N1	1.9686 (14)
Zn1—N2^i^	1.9944 (14)
Zn1—N3	1.9968 (16)
Zn2—O5	2.0266 (12)
Zn2—O4	2.0819 (12)
Zn2—O1	2.1862 (12)

Symmetry code: (i) 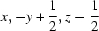.

**Table 2:** Hydrogen-bond geometry (Å, °)

*D*—H⋯*A*	*D*—H	H⋯*A*	*D*⋯*A*	*D*—H⋯*A*
N4—H4*B*⋯O5^ii^	0.86	1.91	2.756 (2)	167

Symmetry code: (ii) 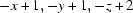.

**Fig. 1.**
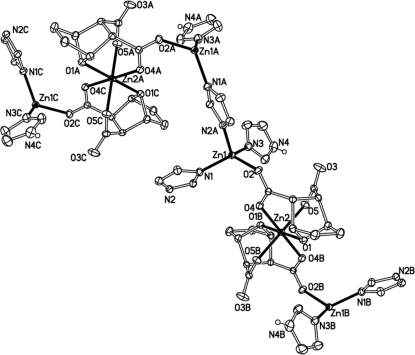
A view of the molecule of the title compound showing the atom-labelling and connectivity of the ZnII atoms. Displacement ellipsoids drawn at the 30% probability level.

## supplementary crystallographic information

### Comment

7-oxabicyclo[2,2,1] heptane-2,3-dicarboxylic anhydride (norcantharidin) derived from cantharidin is a lower toxicity anticancer drug (Shimi *et al.*, 1982). Imidazole is reputed as biocatalyst and biological ligand. Several cobalt complexes of norcantharidin (Wang *et al.*, 1988) and of imidazole (Furenlid *et al.*, 1986; Zhu *et al.*, 2003) have been reported previously.

In the structure of the title compound, the Zn1(II) cation is four-coordinated by three nitrogen atoms of three imidazoles ligands, two of which are deprotonated, and by one carboxylate oxygen atom of the demethylcantharate anion. The deprotonated imidazole rings are responsible for bridging neighbouring Zn1 atoms. The Zn2(II) cation is located on a crystallographic centre of inversion. Two bridge oxygen atoms of two symmetry-related demethylcantharate anions and four carboxylate oxygen atoms give rise to a slightly distorted octahedral ZnO_6_ coordination environment. Each demethylcantharate anion adopts simultaneously a bridging coordination mode (O2 towards Zn1, O4 towards Zn2) and a monodentate coordination mode (through O5 towards Zn2).

The crystal lattice is stabilized through N—H···O hydrogen bonds between the uncoordinated nitrogen atom (N4) of the imidazole molecule and one of the carboxylate oxygen atoms (O3) of the demethylcantharate anion.

### Experimental

7-oxabicyclo[2,2,1] heptane-2,3-dicarboxylic anhydride, zinc acetate and imidazole were dissolved in 15 mL distilled water. The mixture was sealed in a 25 mL Teflon-lined stainless vessel and heated at 443 K for 3 d, then cooled slowly to room temperature. Crystals suitable for X-ray diffraction were obtained.

### Refinement

The H atoms bonded to C and N atoms were positioned geometrically and refined using a riding model [aromatic C—H 0.93 Å, aliphatic C—H = 0.97 (2) Å and N—H = 0.86 Å with U_iso_(H) = 1.2U_eq_(C,N)].

### Crystal data

**Table:**

[Zn_3_(C_8_H_8_O_5_)_2_(C_3_H_3_N_2_)_2_(C_3_H_4_N_2_)_2_]	*F*(000) = 848
*M**_r_* = 834.71	*D*_x_ = 1.718 Mg m^−^^3^
Monoclinic, *P*2_1_/*c*	Mo *K*α radiation, λ = 0.71073 Å
Hall symbol: -P 2ybc	Cell parameters from 5397 reflections
*a* = 7.9993 (1) Å	θ = 1.8–27.6°
*b* = 22.3923 (2) Å	µ = 2.28 mm^−^^1^
*c* = 9.7586 (1) Å	*T* = 296 K
β = 112.633 (1)°	Block, colourless
*V* = 1613.37 (3) Å^3^	0.28 × 0.17 × 0.09 mm
*Z* = 2	

### Data collection

**Table:**

Bruker APEXII CCD diffractometer	3714 independent reflections
Radiation source: fine-focus sealed tube	3197 reflections with *I* > 2σ(*I*)
graphite	*R*_int_ = 0.023
ω scans	θ_max_ = 27.6°, θ_min_ = 1.8°
Absorption correction: multi-scan (*SADABS*; Sheldrick, 1996)	*h* = −10→10
*T*_min_ = 0.629, *T*_max_ = 0.822	*k* = −29→28
13439 measured reflections	*l* = −12→12

### Refinement

**Table:**

Refinement on *F*^2^	Primary atom site location: structure-invariant direct methods
Least-squares matrix: full	Secondary atom site location: difference Fourier map
*R*[*F*^2^ > 2σ(*F*^2^)] = 0.024	Hydrogen site location: inferred from neighbouring sites
*wR*(*F*^2^) = 0.060	H-atom parameters constrained
*S* = 1.03	*w* = 1/[σ^2^(*F*_o_^2^) + (0.0306*P*)^2^ + 0.4382*P*] where *P* = (*F*_o_^2^ + 2*F*_c_^2^)/3
3714 reflections	(Δ/σ)_max_ = 0.001
223 parameters	Δρ_max_ = 0.36 e Å^−^^3^
0 restraints	Δρ_min_ = −0.28 e Å^−^^3^

### Special details

**Table:**

Geometry. All esds (except the esd in the dihedral angle between two l.s. planes) are estimated using the full covariance matrix. The cell esds are taken into account individually in the estimation of esds in distances, angles and torsion angles; correlations between esds in cell parameters are only used when they are defined by crystal symmetry. An approximate (isotropic) treatment of cell esds is used for estimating esds involving l.s. planes.
Refinement. Refinement of *F*^2^ against ALL reflections. The weighted *R*-factor wR and goodness of fit *S* are based on *F*^2^, conventional *R*-factors *R* are based on *F*, with *F* set to zero for negative *F*^2^. The threshold expression of *F*^2^ > σ(*F*^2^) is used only for calculating *R*-factors(gt) etc. and is not relevant to the choice of reflections for refinement. *R*-factors based on *F*^2^ are statistically about twice as large as those based on *F*, and *R*- factors based on ALL data will be even larger.

### Fractional atomic coordinates and isotropic or equivalent isotropic displacement parameters (Å^2^)

**Table:**

	*x*	*y*	*z*	*U*_iso_*/*U*_eq_	
Zn1	0.02705 (3)	0.327653 (8)	0.68480 (2)	0.02488 (7)	
Zn2	0.0000	0.5000	1.0000	0.02427 (8)	
C1	0.5856 (3)	0.35035 (11)	0.8423 (3)	0.0508 (6)	
H1A	0.7015	0.3415	0.8476	0.061*	
C2	0.4334 (3)	0.31994 (10)	0.7673 (3)	0.0471 (5)	
H2A	0.4259	0.2860	0.7103	0.057*	
C3	0.3601 (3)	0.39293 (9)	0.8744 (2)	0.0391 (5)	
H3A	0.2943	0.4196	0.9073	0.047*	
C4	−0.1719 (3)	0.18968 (8)	0.8571 (2)	0.0333 (4)	
H4A	−0.2451	0.1565	0.8488	0.040*	
C5	−0.1783 (3)	0.22588 (8)	0.7447 (2)	0.0325 (4)	
H5A	−0.2566	0.2218	0.6459	0.039*	
C6	0.0280 (3)	0.25794 (8)	0.94487 (19)	0.0293 (4)	
H6A	0.1206	0.2810	1.0110	0.035*	
C7	−0.1093 (2)	0.44234 (7)	0.70651 (18)	0.0222 (3)	
C8	−0.1796 (2)	0.50314 (7)	0.64085 (19)	0.0230 (3)	
H8A	−0.2375	0.5001	0.5325	0.028*	
C9	−0.3146 (2)	0.52910 (8)	0.70277 (19)	0.0267 (4)	
H9A	−0.3937	0.4991	0.7198	0.032*	
C10	−0.4169 (3)	0.58168 (8)	0.6063 (2)	0.0364 (4)	
H10A	−0.5217	0.5927	0.6277	0.044*	
H10B	−0.4555	0.5726	0.5015	0.044*	
C11	−0.2716 (3)	0.63123 (8)	0.6539 (2)	0.0395 (5)	
H11A	−0.2465	0.6459	0.5702	0.047*	
H11B	−0.3078	0.6644	0.7003	0.047*	
C12	−0.1090 (3)	0.59822 (8)	0.7649 (2)	0.0296 (4)	
H12A	−0.0177	0.6248	0.8336	0.036*	
C13	−0.0311 (2)	0.55369 (7)	0.68508 (19)	0.0250 (4)	
H13A	−0.0282	0.5722	0.5950	0.030*	
C14	0.1603 (2)	0.53377 (8)	0.78551 (19)	0.0265 (4)	
N1	−0.0501 (2)	0.26968 (6)	0.80047 (16)	0.0281 (3)	
N2	−0.0394 (2)	0.20990 (6)	0.98546 (16)	0.0288 (3)	
N3	0.2899 (2)	0.34675 (7)	0.78774 (18)	0.0333 (4)	
N4	0.5372 (2)	0.39636 (8)	0.9085 (2)	0.0429 (4)	
H4B	0.6087	0.4232	0.9632	0.051*	
O1	−0.19478 (17)	0.55896 (5)	0.83772 (13)	0.0271 (3)	
O2	−0.09952 (19)	0.40346 (5)	0.61684 (13)	0.0326 (3)	
O3	0.2753 (2)	0.52710 (8)	0.73430 (17)	0.0515 (4)	
O4	−0.06572 (18)	0.43224 (5)	0.84191 (13)	0.0293 (3)	
O5	0.19131 (16)	0.52692 (6)	0.92484 (13)	0.0299 (3)	

### Atomic displacement parameters (Å^2^)

**Table:**

	*U*^11^	*U*^22^	*U*^33^	*U*^12^	*U*^13^	*U*^23^
Zn1	0.03053 (12)	0.02231 (11)	0.02322 (11)	−0.00257 (8)	0.01193 (9)	−0.00100 (7)
Zn2	0.02831 (15)	0.02813 (15)	0.01822 (14)	−0.00145 (11)	0.01100 (12)	−0.00224 (10)
C1	0.0314 (11)	0.0607 (14)	0.0607 (15)	−0.0008 (10)	0.0180 (11)	−0.0062 (12)
C2	0.0400 (12)	0.0476 (13)	0.0559 (14)	0.0009 (9)	0.0209 (11)	−0.0142 (10)
C3	0.0339 (10)	0.0342 (10)	0.0435 (12)	0.0002 (8)	0.0085 (9)	−0.0071 (9)
C4	0.0360 (10)	0.0285 (9)	0.0353 (11)	−0.0069 (8)	0.0135 (9)	0.0026 (8)
C5	0.0348 (10)	0.0339 (10)	0.0254 (9)	−0.0060 (8)	0.0077 (8)	0.0002 (7)
C6	0.0355 (10)	0.0247 (9)	0.0267 (9)	−0.0021 (7)	0.0107 (8)	0.0007 (7)
C7	0.0217 (8)	0.0222 (8)	0.0228 (9)	−0.0034 (6)	0.0088 (7)	−0.0027 (6)
C8	0.0265 (9)	0.0228 (8)	0.0185 (8)	−0.0016 (6)	0.0075 (7)	−0.0007 (6)
C9	0.0256 (9)	0.0266 (9)	0.0275 (9)	−0.0022 (7)	0.0096 (7)	−0.0005 (7)
C10	0.0303 (10)	0.0353 (10)	0.0400 (11)	0.0077 (8)	0.0095 (9)	0.0041 (8)
C11	0.0435 (12)	0.0266 (10)	0.0465 (12)	0.0070 (8)	0.0154 (10)	0.0055 (8)
C12	0.0342 (10)	0.0217 (8)	0.0334 (10)	−0.0031 (7)	0.0134 (8)	−0.0021 (7)
C13	0.0287 (9)	0.0253 (8)	0.0229 (8)	−0.0028 (7)	0.0121 (7)	0.0023 (6)
C14	0.0271 (9)	0.0272 (9)	0.0270 (9)	−0.0059 (7)	0.0124 (8)	−0.0009 (7)
N1	0.0341 (8)	0.0252 (7)	0.0249 (8)	−0.0016 (6)	0.0114 (7)	0.0028 (6)
N2	0.0376 (9)	0.0254 (8)	0.0256 (8)	0.0013 (6)	0.0148 (7)	0.0035 (6)
N3	0.0301 (8)	0.0309 (8)	0.0371 (9)	−0.0032 (7)	0.0110 (7)	−0.0046 (7)
N4	0.0319 (9)	0.0396 (9)	0.0461 (11)	−0.0091 (7)	0.0028 (8)	−0.0045 (8)
O1	0.0316 (7)	0.0272 (6)	0.0250 (6)	0.0010 (5)	0.0136 (5)	−0.0020 (5)
O2	0.0497 (8)	0.0243 (6)	0.0235 (6)	0.0059 (5)	0.0137 (6)	−0.0031 (5)
O3	0.0368 (8)	0.0864 (12)	0.0406 (9)	0.0114 (8)	0.0249 (7)	0.0152 (8)
O4	0.0439 (8)	0.0233 (6)	0.0203 (6)	0.0005 (5)	0.0120 (6)	−0.0004 (5)
O5	0.0258 (6)	0.0419 (7)	0.0226 (6)	−0.0049 (5)	0.0099 (5)	−0.0012 (5)

### Geometric parameters (Å, °)

**Table:**

Zn1—O2	1.9570 (12)	C6—H6A	0.9300
Zn1—N1	1.9686 (14)	C7—O4	1.251 (2)
Zn1—N2^i^	1.9944 (14)	C7—O2	1.2578 (19)
Zn1—N3	1.9968 (16)	C7—C8	1.518 (2)
Zn2—O5^ii^	2.0266 (12)	C8—C9	1.540 (2)
Zn2—O5	2.0266 (12)	C8—C13	1.576 (2)
Zn2—O4	2.0819 (12)	C8—H8A	0.9800
Zn2—O4^ii^	2.0819 (12)	C9—O1	1.459 (2)
Zn2—O1^ii^	2.1862 (12)	C9—C10	1.533 (2)
Zn2—O1	2.1862 (12)	C9—H9A	0.9800
C1—C2	1.340 (3)	C10—C11	1.543 (3)
C1—N4	1.350 (3)	C10—H10A	0.9700
C1—H1A	0.9300	C10—H10B	0.9700
C2—N3	1.376 (3)	C11—C12	1.525 (3)
C2—H2A	0.9300	C11—H11A	0.9700
C3—N3	1.317 (2)	C11—H11B	0.9700
C3—N4	1.327 (3)	C12—O1	1.457 (2)
C3—H3A	0.9300	C12—C13	1.537 (2)
C4—C5	1.349 (3)	C12—H12A	0.9800
C4—N2	1.369 (2)	C13—C14	1.533 (2)
C4—H4A	0.9300	C13—H13A	0.9800
C5—N1	1.371 (2)	C14—O3	1.213 (2)
C5—H5A	0.9300	C14—O5	1.294 (2)
C6—N1	1.329 (2)	N2—Zn1^iii^	1.9944 (14)
C6—N2	1.330 (2)	N4—H4B	0.8600
			
O2—Zn1—N1	122.06 (6)	C10—C9—C8	109.78 (15)
O2—Zn1—N2^i^	97.25 (6)	O1—C9—H9A	113.8
N1—Zn1—N2^i^	104.86 (6)	C10—C9—H9A	113.8
O2—Zn1—N3	106.98 (6)	C8—C9—H9A	113.8
N1—Zn1—N3	110.76 (7)	C9—C10—C11	101.84 (15)
N2^i^—Zn1—N3	114.52 (7)	C9—C10—H10A	111.4
O5^ii^—Zn2—O5	180.000 (1)	C11—C10—H10A	111.4
O5^ii^—Zn2—O4	92.34 (5)	C9—C10—H10B	111.4
O5—Zn2—O4	87.66 (5)	C11—C10—H10B	111.4
O5^ii^—Zn2—O4^ii^	87.66 (5)	H10A—C10—H10B	109.3
O5—Zn2—O4^ii^	92.34 (5)	C12—C11—C10	101.71 (15)
O4—Zn2—O4^ii^	180.0	C12—C11—H11A	111.4
O5^ii^—Zn2—O1^ii^	89.15 (5)	C10—C11—H11A	111.4
O5—Zn2—O1^ii^	90.85 (5)	C12—C11—H11B	111.4
O4—Zn2—O1^ii^	90.17 (4)	C10—C11—H11B	111.4
O4^ii^—Zn2—O1^ii^	89.83 (4)	H11A—C11—H11B	109.3
O5^ii^—Zn2—O1	90.85 (5)	O1—C12—C11	101.88 (15)
O5—Zn2—O1	89.15 (5)	O1—C12—C13	102.39 (13)
O4—Zn2—O1	89.83 (4)	C11—C12—C13	110.85 (16)
O4^ii^—Zn2—O1	90.17 (4)	O1—C12—H12A	113.5
O1^ii^—Zn2—O1	180.0	C11—C12—H12A	113.5
C2—C1—N4	106.4 (2)	C13—C12—H12A	113.5
C2—C1—H1A	126.8	C14—C13—C12	111.41 (14)
N4—C1—H1A	126.8	C14—C13—C8	115.37 (13)
C1—C2—N3	109.4 (2)	C12—C13—C8	101.21 (13)
C1—C2—H2A	125.3	C14—C13—H13A	109.5
N3—C2—H2A	125.3	C12—C13—H13A	109.5
N3—C3—N4	110.88 (18)	C8—C13—H13A	109.5
N3—C3—H3A	124.6	O3—C14—O5	123.32 (17)
N4—C3—H3A	124.6	O3—C14—C13	120.10 (16)
C5—C4—N2	108.61 (16)	O5—C14—C13	116.54 (15)
C5—C4—H4A	125.7	C6—N1—C5	104.69 (15)
N2—C4—H4A	125.7	C6—N1—Zn1	127.96 (13)
C4—C5—N1	108.57 (16)	C5—N1—Zn1	126.56 (12)
C4—C5—H5A	125.7	C6—N2—C4	104.75 (14)
N1—C5—H5A	125.7	C6—N2—Zn1^iii^	130.16 (12)
N1—C6—N2	113.37 (16)	C4—N2—Zn1^iii^	125.09 (12)
N1—C6—H6A	123.3	C3—N3—C2	105.22 (17)
N2—C6—H6A	123.3	C3—N3—Zn1	126.68 (14)
O4—C7—O2	122.91 (15)	C2—N3—Zn1	127.52 (14)
O4—C7—C8	121.03 (15)	C3—N4—C1	108.14 (17)
O2—C7—C8	116.06 (15)	C3—N4—H4B	125.9
C7—C8—C9	112.03 (14)	C1—N4—H4B	125.9
C7—C8—C13	114.24 (14)	C12—O1—C9	96.04 (12)
C9—C8—C13	100.96 (13)	C12—O1—Zn2	112.31 (10)
C7—C8—H8A	109.8	C9—O1—Zn2	114.50 (9)
C9—C8—H8A	109.8	C7—O2—Zn1	121.81 (11)
C13—C8—H8A	109.8	C7—O4—Zn2	122.65 (10)
O1—C9—C10	102.25 (14)	C14—O5—Zn2	123.34 (11)
O1—C9—C8	102.16 (13)		
			
N4—C1—C2—N3	−0.5 (3)	C1—C2—N3—C3	0.3 (3)
N2—C4—C5—N1	−0.1 (2)	C1—C2—N3—Zn1	171.94 (17)
O4—C7—C8—C9	−42.3 (2)	O2—Zn1—N3—C3	38.44 (19)
O2—C7—C8—C9	137.10 (16)	N1—Zn1—N3—C3	−96.74 (18)
O4—C7—C8—C13	71.7 (2)	N2^i^—Zn1—N3—C3	144.95 (17)
O2—C7—C8—C13	−108.87 (17)	O2—Zn1—N3—C2	−131.53 (18)
C7—C8—C9—O1	86.39 (15)	N1—Zn1—N3—C2	93.29 (19)
C13—C8—C9—O1	−35.59 (15)	N2^i^—Zn1—N3—C2	−25.0 (2)
C7—C8—C9—C10	−165.65 (14)	N3—C3—N4—C1	−0.4 (3)
C13—C8—C9—C10	72.37 (16)	C2—C1—N4—C3	0.5 (3)
O1—C9—C10—C11	33.06 (18)	C11—C12—O1—C9	57.35 (15)
C8—C9—C10—C11	−74.84 (18)	C13—C12—O1—C9	−57.40 (14)
C9—C10—C11—C12	2.0 (2)	C11—C12—O1—Zn2	176.96 (11)
C10—C11—C12—O1	−36.52 (18)	C13—C12—O1—Zn2	62.21 (13)
C10—C11—C12—C13	71.83 (19)	C10—C9—O1—C12	−55.89 (15)
O1—C12—C13—C14	−88.49 (16)	C8—C9—O1—C12	57.76 (14)
C11—C12—C13—C14	163.49 (15)	C10—C9—O1—Zn2	−173.78 (11)
O1—C12—C13—C8	34.66 (16)	C8—C9—O1—Zn2	−60.13 (13)
C11—C12—C13—C8	−73.36 (17)	O5^ii^—Zn2—O1—C12	170.75 (10)
C7—C8—C13—C14	0.5 (2)	O5—Zn2—O1—C12	−9.25 (10)
C9—C8—C13—C14	120.96 (15)	O4—Zn2—O1—C12	−96.91 (10)
C7—C8—C13—C12	−119.84 (15)	O4^ii^—Zn2—O1—C12	83.09 (10)
C9—C8—C13—C12	0.57 (16)	O5^ii^—Zn2—O1—C9	−81.07 (11)
C12—C13—C14—O3	−140.91 (18)	O5—Zn2—O1—C9	98.93 (11)
C8—C13—C14—O3	104.4 (2)	O4—Zn2—O1—C9	11.27 (11)
C12—C13—C14—O5	36.7 (2)	O4^ii^—Zn2—O1—C9	−168.73 (11)
C8—C13—C14—O5	−77.90 (19)	O4—C7—O2—Zn1	−11.7 (2)
N2—C6—N1—C5	0.4 (2)	C8—C7—O2—Zn1	168.88 (11)
N2—C6—N1—Zn1	−169.85 (12)	N1—Zn1—O2—C7	61.89 (16)
C4—C5—N1—C6	−0.2 (2)	N2^i^—Zn1—O2—C7	174.51 (14)
C4—C5—N1—Zn1	170.27 (13)	N3—Zn1—O2—C7	−67.06 (15)
O2—Zn1—N1—C6	−105.18 (16)	O2—C7—O4—Zn2	159.29 (13)
N2^i^—Zn1—N1—C6	146.15 (16)	C8—C7—O4—Zn2	−21.3 (2)
N3—Zn1—N1—C6	22.12 (17)	O5^ii^—Zn2—O4—C7	124.12 (14)
O2—Zn1—N1—C5	86.57 (16)	O5—Zn2—O4—C7	−55.88 (14)
N2^i^—Zn1—N1—C5	−22.11 (17)	O1^ii^—Zn2—O4—C7	−146.73 (14)
N3—Zn1—N1—C5	−146.14 (15)	O1—Zn2—O4—C7	33.27 (14)
N1—C6—N2—C4	−0.5 (2)	O3—C14—O5—Zn2	−150.62 (16)
N1—C6—N2—Zn1^iii^	179.67 (12)	C13—C14—O5—Zn2	31.8 (2)
C5—C4—N2—C6	0.3 (2)	O4—Zn2—O5—C14	47.74 (14)
C5—C4—N2—Zn1^iii^	−179.80 (13)	O4^ii^—Zn2—O5—C14	−132.26 (14)
N4—C3—N3—C2	0.1 (2)	O1^ii^—Zn2—O5—C14	137.88 (14)
N4—C3—N3—Zn1	−171.69 (14)	O1—Zn2—O5—C14	−42.12 (14)

Symmetry codes: (i) *x*, −*y*+1/2, *z*−1/2; (ii) −*x*, −*y*+1, −*z*+2; (iii) *x*, −*y*+1/2, *z*+1/2.

### Hydrogen-bond geometry (Å, °)

**Table:**

*D*—H···*A*	*D*—H	H···*A*	*D*···*A*	*D*—H···*A*
N4—H4B···O5^iv^	0.86	1.91	2.756 (2)	167

Symmetry codes: (iv) −*x*+1, −*y*+1, −*z*+2.
